# Gene conversion restores selfing in experimentally evolving *C. elegans* populations with *fog-2* loss-of-function mutation

**DOI:** 10.17912/micropub.biology.000569

**Published:** 2022-05-19

**Authors:** Weronika Antoł, Joanna K. Palka, Karolina Sychta, Katarzyna Dudek, Zofia M. Prokop

**Affiliations:** 1 Jagiellonian University in Kraków, Institute of Environmental Sciences, Poland; 2 Polish Academy of Sciences, Institute of Systematics and Evolution of Animals, Poland

## Abstract

We have discovered a new case of gene conversion restoring ability of self-fertilization in obligatory outcrossing *Caenorhabditis elegans* populations. The *fog-2(q71)* mutation, used to transform the nematodes’ mating system from mostly self-fertilization to obligatory outcrossing, was spontaneously removed by replacing a fragment of *fog-2* gene with a fragment of its paralog, *ftr-1* . This has occurred spontaneously in experimental evolution with large populations, evolving with *fog-2(q71)* mutation for over a hundred generations, without addition mutagens or other factors promoting mutation accumulation. A converted *fog-2* allele restoring hermaphrodite sperm production was detected in five experimental populations. This raises the question about stability of obligatory outcrossing populations in long-term experiments.

**Figure 1.**
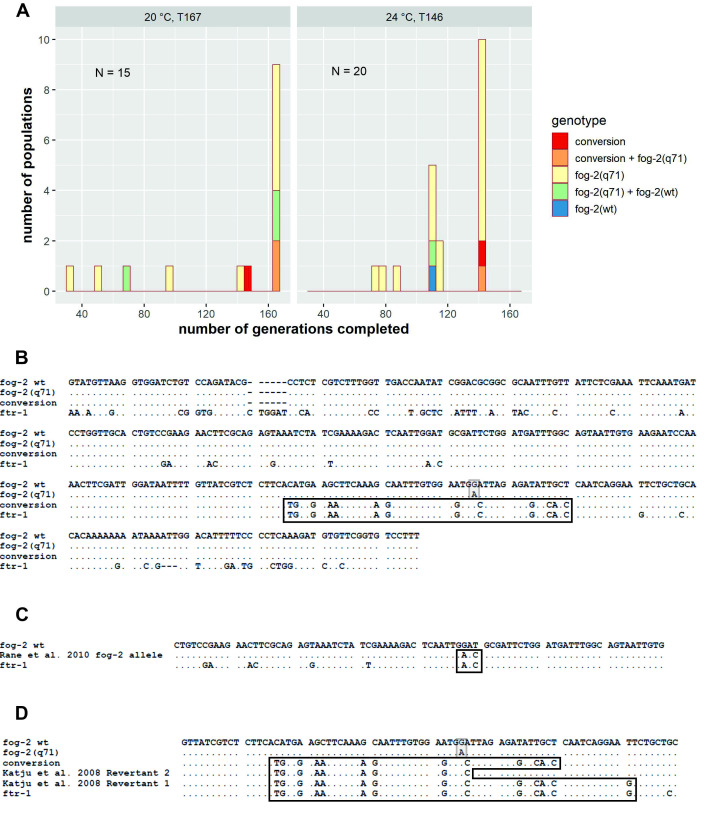
**A** . Genotypes of experimental populations in 20 °C (left) and 24 °C (right). Population genotypes in *fog-2* are depicted using different colours. In 20 °C, all populations were screened at transfer 167 (15 populations) and in 24 °C – at transfer 146 (20 populations). However, transfer number is not the same as number of generations completed by particular populations - the populations were being transferred simultaneously and the transfer number was incrementing for the whole experiment but the number of generations completed was noted for each population individually. Due to cases when during the experiment re-starting of some populations from frozen stock was needed, number of generations for a particular populations was lower than the transfer number. Therefore, although all the bars present populations that were sampled at the same experimental transfer (167 in 20 °C, 146 in 24 °C), they differ in the number of generations completed by that time. **B** . Aligned fragments of *fog-2* and *ftr-1* genes in *Caenorhabditis elegans.* Gene fragments sequenced in our study encompass the *fog-2(q71)* mutation site with up- and downstream flanking regions of ca. 260 and 100 nucleotides, respectively. " *fog-2 wt* " is a fragment of wild type sequence of *fog-2* . " *fog-2(q71)* " is a sequence of *fog-2* with loss-of-function mutation G ⟶ A (indicated with a shaded box), resulting in obligatory outcrossing. "Conversion" is a sequence of *fog-2* from a population with *fog-2(q71)* mutation, which has restored the ability of self-fertilization by reparation of the mutated site with a fragment of *ftr-1* . " *ftr-1* " is a fragment of *ftr-1* , a paralog of *fog-2* . The minimal region of conversion (45 nucleotides) is indicated with a box in bold. **C.** An example of *fog-2* allele resulting from gene conversion with *ftr-1* found in nature (Rane et al. 2010), located ca. 100 nucleotides upstream to the site of *fog-2(q71)* mutation shown in A). This variant was found to have a detrimental effect on fecundity. **D** . Comparison of the conversion regions (box in bold) observed in our study in *fog-2* ("conversion", minimum 45 bp) and in the study of Katju et al. 2008 ("Revertant 2", min. 31 bp and "Revertant 1", min. 56 bp). Shaded box indicates the position of *fog-2(q71)* point mutation.

## Description

*Caenorhabditis elegans* is a model species, increasingly used in experimental evolution studies to investigate such major topics as: maintenance of genetic variation, host-pathogen interaction and coevolution, mutations, life history, evolution of reproductive systems, sexual selection (Gray & Cutter, 2014; Teotónio, Estes, Phillips, & Baer, 2017). Its reproductive system in the wild, known as androdioecy, involves mostly self-fertilization of hermaphrodites and occasionally outcrossing with males, which are generally rare (Stewart & Phillips, 2002). This system can be experimentally changed to dioecy, i.e., obligatory outcrossing, through genetic manipulations (see Table I in Anderson, Morran, & Phillips, 2010; Gray & Cutter, 2014)

Experimental populations of obligatory outcrossers enable studying a wide range of evolutionary questions related to the evolution of reproductive systems and sexual traits (Morran et al., 2009, Anderson et al., 2010, more examples reviewed by Teotónio et al., 2017). Therefore, maintenance of obligatorily outcrossing strains in laboratory conditions is of interest for many research fields. However, fitness of outcrossing populations can be expected to be significantly lower than in selfing populations as males do not produce offspring directly (Anderson et al., 2010), which makes outcrossing populations prone to invasion of self-fertilizing hermaphrodites (Theologidis et al., 2014). One of the most commonly used methods is blocking sperm production in hermaphrodites by knocking down the gene *fog-2* , required for spermatogenesis in hermaphrodites but not in males (Schedl & Kimble, 1988). Hermaphrodites are thus transformed into functional females. Populations fixed for the loss-of function mutation in *fog-2(q71)* reproduce exclusively via outcrossing. However, it has been shown experimentally that mutations in several different genes in *fog-2(q71)* -phenotype animals can restore the wild-type phenotype (Francis et al., 1995; Maine et al., 2004; Nayak et al., 2005; Schedl & Kimble, 1988). Spontaneous cases of gene conversion restoring the ability of *fog-2* mutated populations to self-fertilize have been reported in one study (Katju et al., 2008). There, the researchers have found that a fragment of *fog-2* including the loss-of-function mutation was replaced with a fragment of *ftr-1,* a paralog of *fog-2* of unknown function (Clifford et al., 2000; Rane et al., 2010), resulting in restored functionality of *fog-2* .

Here, we describe a new case of gene conversion between *ftr-1* and *fog-2* restoring *fog-2* function, which we have discovered in experimental evolution of large (ca 10 000 individuals) *C. elegans* populations. With this finding we conclude that the stability of the obligatorily outcrossing genotype should be carefully monitored during long-term evolution studies involving *fog-2(q71)* populations.

In this study, we have screened *fog-2(q71)* populations of *C. elegans* evolving in 20 °C (optimal temperature; 15 populations) or 24 °C (elevated temperature; 20 populations) for their genotype in *fog-2 (i.e., fog-2(q71) or fog-2-wild type).* The screening was performed after 167 transfers for the populations in 20 °C and after 146 transfers for the populations in 24 °C (the exact number of generations completed by each population is presented in Figure 1A). From each population, a sample of several hundreds of animals was pooled and sequenced at two loci: *fog-2* (to check the mutation site and its surrounding) and *ftr-1* (as a potential donor sequence for conversion). Along the evolved *fog-2(q71)* populations, we also sequenced the same loci from wild type (N2) populations as controls.

Among all samples, four different sequences were obtained in total, three of which were identical to the known reference sequences: *ftr-1, fog-2* wild-type or *­fog-2(q71)* gene fragments. The fourth sequence we concluded to be a new *fog-2* allele, a result of conversion between *fog-2* and *ftr-1* as it was identical to *fog-2* reference sequence over most of the sites, except only the region of 45 nucleotides encompassing mutation site in *­fog-2(q71)* reference, where it was identical to *ftr-1* (see Figure 1 B). This converted *fog-2* allele was found in five populations (Figure 1 A). In two of them (one in 20 °C, generation 147 and one in 24 °C, generation 141) it was the only *fog-2* allele detected in the sample, in three (two in 20 °C, generation 165 and one in 24 °C, generation 142) it was coexisting with the *­fog-2(q71)* allele. Phenotypically, in visual screening of these populations, the proportion of males was strikingly lower than 0.5; in some cases virtually 0. These populations were still reproducing, indicating that sperm production was indeed restored in hermaphrodites possessing the converted allele. Furthermore, wild type *fog-2* allele was also found in five evolved populations (in one population it was the only *fog-2* allele detected, in four populations – coexisting with the *fog-2(q71)* allele; see Figure 1A), indicating cross-contamination (discussed below) from wild type populations which were evolving alongside the outcrossing ones in our experiment. Alternatively, the wild-type *fog-2* allele could have remained, at trace frequency, during the construction of ancestral ‘fog’ populations. However, we consider this to be highly unlikely, because *fog-2(q71)* introgression was followed by 10 generations of brother-sister inbreeding, with careful screening for *fog-2* wild type vs. *q71* phenotypes performed every generation.

The first study reporting spontaneous restoring of *fog-2* function through gene conversion with *ftr-1* (Katju et al., 2008) detected two independent cases among 74 evolving populations. One of them occurred in the experimental phase of repeated bottlenecks and knockdown of the mismatch repair gene *msh-2* , another one – in the phase without these factors that enhance mutation accumulation. In our case, population census size was kept in high numbers throughout the experiment (ca. 10 000 indviduals) to allow higher diversity. The identity of the converted fragment in all the cases from our experiment raises question about the independence of conversion events, especially given the cross-contamination cases we detected. However, cross-contaminations are highly unlikely between experimental temperatures, since populations evolving at 20 °C and 24 °C are being transferred separately and kept at separate incubators. Thus, we conclude that at least two independent conversion events have occurred in our populations: at least one in each temperature. It should also be noted that what is identical in all the conversion cases detected in our study, is the minimal region of conversion, which consists of 45 nucleotides and begins in the same position of *fog-2* as in the experiment of Katju et al. (2008), although the end is not identical to any of the two cases described there, which were 31 and 56 nucleotides long (Figure 1 D). The actual conversion region in our populations might be longer as well as may vary between cases which would remain undetected as the minimal converted region is surrounded by nucleotide positions which are invariable between *ftr-1* and *fog-2* (Figure 1 B). This is also why the actual beginning of the converted fragment discovered in our study is not necessarily in the same position as in sequences obtained by (Katju et al., 2008), although the minimal conversion region starts in the same position in all the cases (Figure 1 D).

The converted variant was apparently so beneficial that it was easily spreading in the populations where it was found, which is in line with observations of *C. elegans* experimental populations by Stewart & Phillips (2002) that obligatory outcrossers are rapidly being outcompeted by hermaphrodites and of Chasnov & Chow (2002) who showed that a mutation increasing males frequency is strongly selected against. However, in the study of (Theologidis et al., 2014) self-fertilizing hermaphrodites easily spread in a population of outcrossers but only in harsh conditions (elevated salt concentration) and not in the optimal ones.

From the point of view of researchers using obligatorily outcrossing *fog-2* –mutated populations for experimental evolution, it should be noted that the mutation may be lost spontaneously, changing the desired experimental setup. The prevalence of such events cannot be assessed precisely based on the data available to us, however it appears to be non-negligible. Conversion events between *fog-2* and *ftr-1* were also detected in natural *C. elegans* populations, actually explaining 22-34% of diversity in these genes and potentially impacting fitness by influencing sperm production in hermaphrodites (Rane et al., 2010). For example, one of the converted *fog-2* allele (Figure 1 C) which causes replacement of lysine to asparagine at position 49, was found to decrease brood size by 14% on average.

In conclusion, during long-term evolutionary experiments using *fog-2 -* mutated populations as obligatory outcrossing lines, regular screening should be implemented to detect cases where the reproductive system has reverted to wild type. This can be done at several levels. Visual screening for the proportion of males allows quick and labour-efficient distinguishing between the obligatorily outcrossing populations ( *ca.* 50% males) and the self-fertilizing ones (virtually no males present, at least for N2-background populations), although the precision of such examination is too low to detect early stages of transition between these systems (i.e., beginning of invasion of self-fertilizing hermaphrodites in the population) since the change in male proportion will be gradual. Another way is to randomly sample young females (L4, when sex determination is already possible, but the animals are not yet fertilized) and place them on separate dishes to see if offspring occurs, which in this case would be an effect of selfing. This method requires a trade-off between amount of labour (the number of hermaphrodites sampled) and the aimed precision. Finally, a regular molecular screening can be performed, where a substantial proportion of a population is pooled in one sample, allowing to detect altered variants with higher sensitivity. However, while sequencing *fog-2(lf)* and *ftr-1* fragments allows to detect gene conversion, one should still be aware of possible compensatory mutations in other genes (Schedl & Kimble, 1988; Francis et al., 1995; Maine et al., 2004; Nayak et al., 2005). Thus, combination of several methods would be a recommended way to validate population genotype.

## Methods

Obligatorily outcrossing base populations were created by introgression of *fog-2(q71)* mutation from JK574 strain onto the N2 genetic background (10 cycles of introgression followed by 10 generations of brother-sister inbreeding; for detailed description of introgression, see Plesnar-Bielak et al. 2017; the procedure was implemented following Teotònio et al. 2012). From this base, we derived replicate populations which since then have been evolving independently as a part of an ongoing experimental evolution project with two environmental treatments: control (20 °C) and high temperature (24 °C). Census population sizes have been maintained at ca. 10 000 individuals. Transfers were performed by washing a plate with S Basal solution (Stiernagle, 2006) and using filters with 15 µm eyelets, which only let early larvae through (L1-L2 developmental stage) so that a population can be synchronized. Samples of the ancestral populations and later on from every ca. 12th generation of evolution were also banked by freezing in -80 °C to allow further comparisons. For populations which were lost during the experimental evolution due to cross-contamination or chance events, this banking also allowed re-starting the evolution from its earlier stage (which happened on several occasions in each temperature, cf. Figure 1 A). Screening for the maintenance of the mutated form *fog-2(q71)* allele was performed on 15 control and 20 high temperature populations after 167 and 146 transfers, respectively. The number of generations completed at the time of screening was not equal to the number of transfers due to cases when re-starting of some populations from frozen stock was needed.

DNA extraction samples containing several hundreds of individuals were obtained through washing fragments of each population plate. DNA was extracted using Wizard® Genomic DNA Purification Kit (Promega). We have sequenced fragments of *fog-2* (to check the mutation site and its surrounding) and *ftr-1* (as a potential donor sequence for conversion) (Katju et al., 2008) in the experimental populations and, as control, a wild-type N2 population as well as a *fog-2(q71)* population confirmed in PCR. We have designed one primer pair to amplify both a 390 bp fragment of *fog-2* containing point mutation site and corresponding 393 bp region of *ftr-1* : F-seq: 5’-GACACTGATCCTGGCTTCAA-3’ and R-seq: 5’-AAATCCAGGCTTGCAGTGAG-3’. PCR was performed in 14 µl (1 µl DNA, 1 µl of each primer, 7.5 µl DreamTaq Hot Start DNA Polymerase Master Mix (ThermoScientific) , 3.5 µl H _2_ O) under the following conditions: 4 min of initial denaturation at 95 °C, 34 cycles of [30 s denaturation at 94 °C, 30 s annealing at 60 °C, 60 s elongation at 72 °C ] and additional 5 min annealing at 72 °C. Samples were barcoded with a combination of 6 bp indexes at the 5’ end of forward and reverse primers. Amplification success was assessed in 1.5% agarose gel electrophoresis. Samples were then pooled in proportions estimated by eye based on the intensity of electrophoresis bands. Pools were gel-purified, Illumina adaptors were ligated using NEBNext Ultra II DNA Library Prep Kit for Illumina (NEB) according to the PCR-free protocol obtained from manufacturer, libraries were quantitated with Qubit fluorometer and sequenced on Illumina MiSeq (v3 600 cycles kit). Genotyping was performed using the adjustable clustering method implemented in AmpliSAS (Sebastian et al., 2016). Following clustering of sequence variants within amplicons, we considered only variants with per amplicon frequency > 10.0%. Mean coverage was 850. The obtained sequences were then aligned in BioEdit (Hall, 1999).

## Reagents

**Animals**

**Table:** 

**Strain**	**Genotype**	**Available from**
**N2**	*Caenorhabditis elegans*	CGC
**JK574**	*Caenorhabditis elegans fog-2(q71)*	CGC
**N2_q71**	*Caenorhabditis elegans fog-2(q71)*	Generated through introgression of *fog-2(q71)* mutation from JK574 strain to N2 genetic background. Available at the Jagiellonian University, Institute of Environmental Sciences

**Molecular tools**

**Table:** 

**Primer**	**Sequence (5’-3’)**	**Description**
**F-seq**	GACACTGATCCTGGCTTCAA	Primers to amplify fragments of *fog-2* and *ftr-1* .
**R-seq**	AAATCCAGGCTTGCAGTGAG	
